# Evidence for Divergent Evolution of Growth Temperature Preference in Sympatric *Saccharomyces* Species

**DOI:** 10.1371/journal.pone.0020739

**Published:** 2011-06-02

**Authors:** Paula Gonçalves, Elisabete Valério, Cláudia Correia, João M. G. C. F. de Almeida, José Paulo Sampaio

**Affiliations:** Departamento de Ciências da Vida, Centro de Recursos Microbiológicos (CREM), Faculdade de Ciências e Tecnologia, Universidade Nova de Lisboa, Caparica, Portugal; New York State Department of Health and School of Public Health, University at Albany, United States of America

## Abstract

The genus *Saccharomyces* currently includes eight species in addition to the model yeast *Saccharomyces cerevisiae*, most of which can be consistently isolated from tree bark and soil. We recently found sympatric pairs of *Saccharomyces* species, composed of one cryotolerant and one thermotolerant species in oak bark samples of various geographic origins. In order to contribute to explain the occurrence in sympatry of *Saccharomyces* species, we screened *Saccharomyces* genomic data for protein divergence that might be correlated to distinct growth temperature preferences of the species, using the dN/dS ratio as a measure of protein evolution rates and pair-wise species comparisons. In addition to proteins previously implicated in growth at suboptimal temperatures, we found that glycolytic enzymes were among the proteins exhibiting higher than expected divergence when one cryotolerant and one thermotolerant species are compared. By measuring glycolytic fluxes and glycolytic enzymatic activities in different species and at different temperatures, we subsequently show that the unusual divergence of glycolytic genes may be related to divergent evolution of the glycolytic pathway aligning its performance to the growth temperature profiles of the different species. In general, our results support the view that growth temperature preference is a trait that may have undergone divergent selection in the course of ecological speciation in *Saccharomyces*.

## Introduction

The genus *Saccharomyces* is best known for its most emblematic member, the model and industrial yeast *S. cerevisiae,* but comprises in addition eight closely related species [1]–[3]. Since the beginning of pure culture methods, the repeated isolation of *S. cerevisiae* from wine, beer and other fermented beverages, and the difficulty in finding its truly natural habitats, has lead to the common view that this species was a product of domestication [4]–[6]. Hence, *S. cerevisiae* was thought to have originated at the onset of human driven fermentations, which have been traced back to 5,000–10,000 years ago [7], [8], and to have been essentially confined to the vicinity of artificial fermentation environments ever since. As to the other species of the genus for which significant records are available, *S. bayanus* has been connected to beer [9], *S. uvarum* is often associated with cider and wine fermented at low temperatures [10], [11] and *S. pastorianus* (synonym *S. carlsbergensis*) is used for the production of lager beer [12]. These three species were also regarded as domesticated and since they ferment well at low temperatures, they are often referred to as cryotolerant [13].

Recent studies have challenged the domestication model by providing evidence for a natural habitat of *S. cerevisiae*, the bark of certain oak trees in North America and the soil surrounding them, where persistent populations can be found [14]. Subsequently, other studies have shown that this species is composed of both domesticated and wild populations and that domesticated populations derive from their wild relatives [15]. This progress marked the beginning of a new age for this model organism and its siblings, as basic aspects of their ecology can in principle be tested, paving the way for the vast wealth of knowledge concerning their biology to be examined within an ecological framework [16]. The population structure of *S. cerevisiae* [17]–[19] and of its sibling *S. paradoxus* [20]–[22], a species that is not associated with man-driven fermentations, were extensively studied. Those studies showed that whereas the genetic structure of *S. paradoxus* exhibited a strong influence of geography, a different pattern dominated mostly by ecological factors emerged for *S. cerevisiae* [23], [24]. More recently, the habitats and biogeography of the various species of the genus *Saccharomyces* were investigated with a modified isolation protocol that employed a parallel enrichment step at two temperatures (high and low) to maximize the chances of finding both thermotolerant (e.g. *S. cerevisiae* and *S. paradoxus*) and cryotolerant species [25]. This approach provided evidence that several *Saccharomyces* species co-existed in the same habitat, in some cases even in the same tree in temperate ecosystems in North America, Central Europe and the Mediterranean. Sympatry seemed to be related with preferences of the species for different growth temperatures. For example, *S. cerevisiae* (thermotolerant) was frequently found in association with *S. kudriavzevii*, a cryotolerant species with markedly lower maximum growth temperatures. *S. paradoxus* (thermotolerant) was similarly found in sympatry with *S. uvarum* (cryotolerant) [25]. The coexistence of *S. cerevisiae* and *S. kudriavzevii* was confirmed in other studies that investigated also the Mediterranean ecosystem [26] and in North America a case of sympatry between *S. cerevisiae* and *S. paradoxus* [14] was explained by different thermal growth profiles [27].

Since differences in growth temperature preferences are by far the strongest phenotypic discontinuity between sympatric *Saccharomyces* species, our working hypothesis is that the distinct temperature regimes allow the sympatric partners to explore different (temperature) niches within the same habitat, thereby avoiding competitive exclusion. This impelled us to consider the possibility that this trait may have undergone ecologically based divergent selection in some of the species, in line with a hypothetical ecological speciation scenario [28].

All the species in the genus *Saccharomyces* share physiological characteristics unusual among fungi that consist in a marked preference to ferment sugars when they are present at sufficiently high concentrations, irrespectively of oxygen availability [29], [30]. This typical *Saccharomyces* fermentative life strategy is thought to have evolved after a whole genome duplication (WGD) that occurred approximately 100 MYA ago [31], [32] and was followed by a period of dramatic genome remodeling with massive gene loss erasing unwarranted redundancy [33]. The evolution of post-WGD genome architecture has been extensively studied [32], [34], [35], yielding important insights on several aspects of the fate of genes that remained duplicated. Subfunctionalization [32]–[34], [36]–[38] as well as gene dosage effects [39] seem to have contributed for the retention of genes in duplicate. A significant number of glycolytic genes fall under the latter category, since five of the ten glycolytic reactions maintain WGD duplicates in *S. cerevisiae* without evidence for marked subfunctionalization. An attractive hypothesis was put forward stating that an increase in glycolytic flux following WGD may have provided the basis for the subsequent evolution of the typical *Saccharomyces* strategy, which consists in the rapid conversion of sugars into ethanol, to secure the carbon-source and take advantage of ethanol toxicity for competitors [34], [39], [40].

In the present work we used a comparative genomics approach and publicly available complete genome sequences of five *Saccharomyces* species [41], [42] to search for proteins exhibiting molecular evolution patterns suggestive of divergent selection, when species with different growth temperature preferences are compared. The dN/dS ratio [rate of nonsynonymous substitutions per nonsynonymous site (dN)/ rate of synonymous substitutions per synonymous site (dS)] of the complete *Saccharomyces* ORFeomes was employed as a measure of protein divergence corrected for phylogenetic distance. *Saccharomyces* genomic sequence data has been previously used to determine dN/dS ratios both to detect genes containing the signature of adaptive selection [43]–[45] and to evaluate the extent of co-evolution of functionally related genes [46]. Unlike those investigations, our study was directed towards uncovering protein divergence specifically related to differences in growth temperature preference. We addressed this question by determining the dN/dS ratios of the complete ORFeomes of four *Saccharomyces* species in pair-wise comparisons with *S. cerevisiae* and focused our attention in significant changes in the dN/dS ratios determined between different pairs of ortologous genes. As a result, we identified genes that are uncommonly divergent between *S. cerevisiae* (thermotolerant) and *S. uvarum* (cryotolerant). Among the genes found, some had been previously associated with adaptation to growth at suboptimal temperatures. In addition, one unforeseen functional category encompassing the genes encoding glycolytic enzymes also appeared to be significantly over-represented among those exhibiting higher dN/dS ratios in the comparison between *S. cerevisiae* and *S. uvarum.* This prompted us to examine catabolic fluxes at different temperatures in *Saccharomyces* species with different growth temperature preferences. Our results brought to light species-specific trends, in line with an adaptation of glycolysis to different temperature ranges during *Saccharomyces* evolution and generally support an ecological speciation model.

## Results and Discussion

### Temperature determines the outcome of the competition between sympatric *Saccharomyces* species

**Figure 1:**
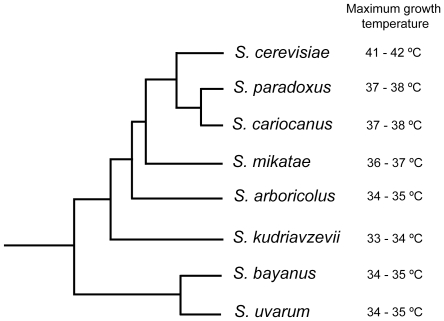
Schematic representation of the phylogeny of the genus *Saccharomyces*. The tree topology combines information from genome-wide studies [47], rDNA sequence data [3], four concatenated nuclear genes [23] and our own sequence analyses. The maximum growth temperature of each species is indicated on the right side (data from this study).

**Figure 2:**
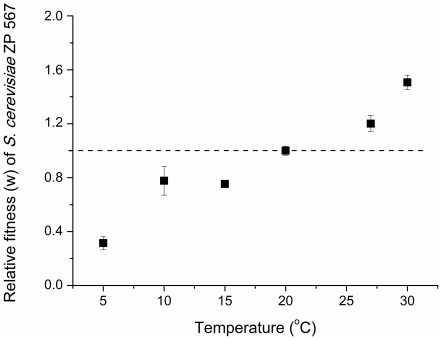
Outcome of competition between sympatric *Saccharomyces* species. Relative fitness of *S. cerevisiae* ZP 567 competing with *S. kudriavzevii* ZP 591 at different temperatures is shown. The relative size of the populations of both species was determined in the beginning and at the end of each co-culture experiment by qRT-PCR using species specific primers, and was used to calculate the relative fitness of each species.

Figure 1 depicts schematically the phylogenetic relationships of the species in the genus *Saccharomyces* and assigns them to the thermotolerant or cryotolerant groups. *S. cerevisiae* and *S. kudriavzevii*, belonging respectively to the former and latter categories, were found in our previous survey to exist in sympatry at several locations in Portugal [25]. In these cases, *S. cerevisiae* was the species isolated when an incubation temperature of 30°C was employed during the enrichment step, while *S. kudriavzevii* was isolated if the same sample was incubated at 10°C. In the same study a similar situation was found for *S. paradoxus* and *S. uvarum* which were sympatric in North America. In order to test the hypothesis that temperature plays a key role in the coexistence of the two populations by contributing for niche divergence and therefore opposing competitive exclusion, we set up a series of competition experiments between a pair of sympatric *S. cerevisiae* (ZP 567) and *S. kudriavzevii* (ZP 591) strains isolated from the same sample of oak bark in Portugal (Table S1A). Both strains were pre-grown separately at the temperature set for each experiment, and were subsequently inoculated in approximately equal amounts in fresh growth medium and co-cultured at a given temperature until stationary phase. These experiments were performed at different temperatures between 5°C and 30°C. The relative size of the two populations was quantified in the beginning and at the end of each co-culture by qReal-Time PCR, using primers specific for each species (Table S1B). The results are shown in Figure 2. Clearly, at the most extreme temperatures one of the species dominates the co-culture in line with its growth temperature preference, while at around 20°C, the two populations remain equilibrated at the end of the co-culture. Similar experiments involving sympatric *S. uvarum* and *S. paradoxus* strains yielded comparable results (Figure S1). Since in the locality of the isolation of *S. cerevisiae* ZP 567 and *S. kudriavzevii* ZP 591 the average low temperatures from November to April range between 5°C and 9°C and from June to September average high temperatures range between 27°C and 32°C, the long-term co-existence of the two populations could be explained by reciprocal fitness variation along a temperature gradient. Hence, these experiments support the hypothesis that temperature is an important ecological determinant modulating the co-existence of two sympatric populations, circadian and / or annual temperature oscillations favoring each species in turn. We searched also for negative effects of one population over the other, like production of killer toxins [16], [48]. Such type of competition by interference was not observed for the two pairs of strains examined (results not shown).

### Protein evolution rates in *Saccharomyces*: cryotolerant *vs* thermotolerant species

**Figure 3:**
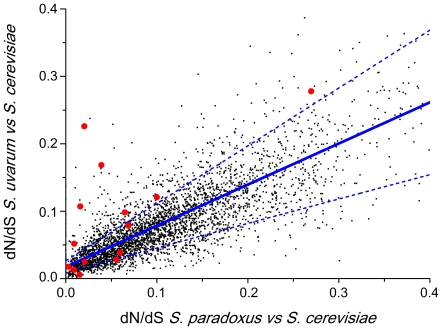
Comparison of dN/dS ratios of *Saccharomyces* orthologous gene pairs in different species comparisons. dN/dS ratios depicted were determined between *S. uvarum* and *S. cerevisiae* (Yaxis) and between *S. paradoxus* and *S. cerevisiae* (Xaxis). Only orthologous gene pairs with dN/dS ratios below 0.4 in both species comparisons are shown (see Figure S2 for a plot containing the complete data set). Each point corresponds to one orthologous gene pair that could be reliably identified in both species comparisons. The regression line is shown in solid blue.The dashed lines add the contribution of the residuals distribution. Points outside these lines may be considered as significantly different from the general trend. The group of orthologs of interest to the present analysis displays higher dN/dS ratios in the comparison between the *S. uvarum* and S. *cerevisiae* genomes than in the comparison between *S. paradoxus* and S. *cerevisiae*, corresponding therefore to data points located above the segment defined by the dashed lines. Data points representing glycolytic genes are highlighted as red circles.

In the course of the adaptation of two putative ancestral *Saccharomyces* sub-populations to distinct niches, as would happen in an ecological speciation scenario, genes important for niche differentiation may have undergone divergent selection [28]. A previous study identified a number of genes exhibiting protein evolutionary rate changes as well as two functional categories that are particularly divergent between *S. cerevisiae* and *S. paradoxus*, but no lineage specific functional biases were uncovered for the other lineages investigated [44]. This study did not focus on any particular phenotype, and its conclusions do not seem to bear a particular relation to the divergence of growth temperature preferences between *Saccharomyces* species. Since this trait is presently the most prominent phenotypic difference between sympatric *Saccharomyces* species, it is also, in our view, the most likely to have experienced divergent selection. To examine this possibility, we looked for genes that exhibit significantly higher dN/dS ratios when measured between *S. cerevisiae* and *S. uvarum*, then when measured between *S. cerevisiae* and *S. paradoxus*. The reasoning behind this is that the first pair of species comprises one thermotolerant and one cryotolerant species, while the second involves two species with similar growth temperature preferences and can therefore be used as a reference. A database of orthologous *Saccharomyces* ORFs was built using the BiDiBlast pipeline [49], which also calculated the dN/dS ratios for all the orthologous gene pairs that could be reliably identified (Table S2). Subsequently, a comparison of the dN/dS ratios determined for *S. cerevisiae* and *S. paradoxus,* with the dN/dS ratios of the same orthologous gene pair determined between *S. cerevisiae* and *S. uvarum* was performed. Identification of genes exhibiting higher dN/dS ratios measured between *S. cerevisiae* and *S. uvarum* was facilitated by plotting the respective values obtained for the complete ORFeome in the two species pairwise comparisons. As shown in Figure 3, the data points corresponding to most of the orthologous gene pairs are concentrated along a diagonal line with a positive slope, a consequence of the fact that most orthologs have similar dN/dS ratios in both species pairwise comparisons. The slope of the regression line is lower than one, indicating that the dN/dS ratios of most ORFs are higher in the *S. cerevisiae*/*S. paradoxus* comparison (Figure 3, Figure S2). This could be an indirect consequence of the greater phylogenetic distance between *S. cerevisiae* and *S. uvarum,* because some genes are likely to exhibit saturated and thus underestimated variation at synonymous sites (dS values) in this pairwise comparison. We subsequently focused on ortholog pairs that fall clearly outside the average distribution, as a result of exhibiting markedly different dN/dS ratios in the two pairwise species comparisons. The genes exhibiting significantly higher dN/dS ratios in the *S. cerevisiae/S.uvarum* comparison correspond to data points located above the interval defined by the dashed lines in Figure 3. It should be noted that this sorting of “accelerated” *vs* “non- accelerated genes” (explained in more detail in the methods section) is quite stringent so that all outliers have experienced a substantial change in dN/dS ratio between the two species pairwise comparisons.

The outliers were subsequently manually identified, purged of residual artifacts caused by incorrect alignments and functionally or structurally related genes were grouped. Because all the species in the analysis are phenotypically similar and phylogenetically relatively close, large differences between the expression levels of orthologous genes are not expected. Hence, codon bias related constraints on the evolution of synonymous sites (dS), which are related to the level of expression [44] are expected to be similar among members of orthologous sets and are therefore unlikely to cause significant artefactual variation in dN/dS ratios in our analysis. Concurrent with this, it has been previously shown that variations in dN/dS ratios among *Saccharomyces* lineages for genes with dS values <1 is mostly due to variation at nonsynonymous sites (dN) [44].

Among the outlier genes, some encode proteins previously shown to be important for growth at suboptimal temperatures in *Saccharomyces cerevisiae*, namely *TIF1* encoding a transcription initiation factor with helicase activity [50], *NSR1*, which is a protein required for rRNA processing [51] and several cell wall mannoproteins *(TIR1, TIR2* and *TIR4;* [52]).

We subsequently explored the possibility that some functional/structural categories might be overrepresented in the accelerated group of genes. To this end categories having multiple genes in the accelerated group were manually examined in more detail. Three categories of genes were found to be significantly accelerated (Table 1). Notably, in these categories, very few”accelerated” genes exhibited dS values>1 that could be indicative of saturation (Table S2), therefore, increased dN/dS ratios in the *S. uvarum/S. cerevisia*e comparison are unlikely to be due to underestimation of dS values in these cases. The first category of “accelerated” genes consisted of cell wall mannoproteins with seven genes displaying accelerated evolution in the *S. cerevisiae/S. uvarum* comparison out of nine genes present in our analysis (Table 1). The second category encompasses the ribosomal components in charge of recruiting translation elongation factors (ribosomal stalk proteins) and subunits of translation elongation factors, suggesting that this process may have evolved as a whole more rapidly during divergence between thermotolerant and cryotolerant species. Finally, at least seven glycolytic genes are also significantly accelerated, which indicates that central carbon metabolism may have diverged more than average in the *S. cerevisiae/S. uvarum* comparison. Changes in dN/dS ratios of glycolytic genes in other species comparisons involving *S. kudriavzevii* and *S. mikatae* were also examined (Table S3). *S. cerevisiae/S. kudriavzevii* represents a second comparison involving a thermotolerant and a cryotolerant species. In this comparison, several glycolytic genes also exhibit considerably higher dN/dS ratios than in the *S. cerevisiae/S.paradoxus* comparison. On the other hand, in the comparison between *S. cerevisiae* and *S. mikatae*, which has intermediate growth temperature preferences (Figure 1) dN/dS ratios of glycolytic genes are generally lower (Table S3).

**Table 1:** Functional categories with elevated dN/dS in the *S. cerevisiae/S. uvarum* comparison

ORF category	Number of genes^a^	p^b^
Cell wall mannoproteins	7 (9;15)	p<0.0001
Ribosomal stalk + Translation elongation factors	6 (8;12)	p = 0.002
Glycolysis	7 (13;17)	p = 0.01

a number of genes with elevated dN/dS in this category (total number of genes in the category present in our analysis; total number of genes in this category).

b probability of observing this or a higher number of genes with elevated dN/dS in our analysis.

These results led us to put forward the hypothesis that the glycolytic pathway may have been under selective pressure to adapt to the different growth temperature regimes during evolution of *Saccharomyces* species. This would be in line with results previously obtained by others, showing that in *S. cerevisiae*, the enzymatic activities of most glycolytic enzymes show very pronounced temperature dependence [53]. As noted for *S. cerevisiae*, losses in glycolytic enzymatic activities are difficult to compensate for with increased expression, because no individual enzyme seems to exert a substantial amount of control over the total flux [53]. This means that overexpression of a large subset or even all the enzymes would be required to increase the flux. Glycolytic genes are already expressed at very high levels so that a significant further increase in expression would be likely to have seriously detrimental effects on fitness. Therefore, it is in our view a plausible hypothesis that the performance of the glycolytic pathway was adjusted by natural selection to the growth temperature preferences of different species and that this might have involved both evolution of the coding regions of glycolytic genes in the different species and concomitant small adjustments at the level of transcriptional regulation. However, it should be noted that at least one glycolytic enzyme in *S. cerevisiae* (Hxk2p) has an important regulatory role in the main glucose repression pathway in addition to its role in glycolysis [54]. Moreover, some glycolytic enzymes have been shown to be located in the cell wall as well as in the cytoplasm of several yeast species [55], [56] and it is so far unclear what their function might be at this location. Therefore, it is pertinent to question whether increased dN/dS ratios of glycolytic genes are actually linked with operation of the glycolytic pathway or if, alternatively, other established or putative secondary functions of glycolytic enzymes may pertain to the pattern of evolution of these genes. We subsequently proceeded to evaluate the plausibility of these alternate possibilities by measuring the total glycolytic flux at different temperatures in *Saccharomyces* species with different growth temperature preferences.

### Species-specific effects of temperature on glycolytic flux

**Figure 4:**
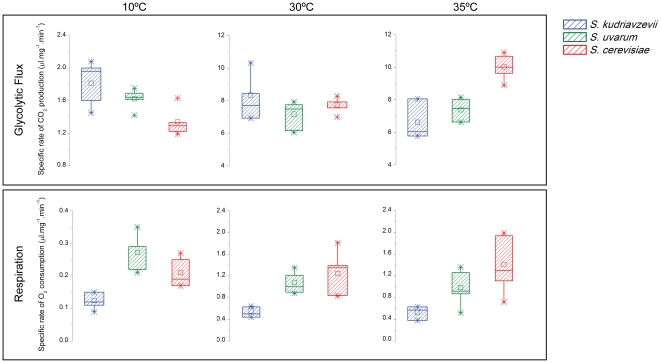
Glycolytic and respiratory fluxes at different temperatures. Fluxes were measured at10°, 30° and 35°C in *S. kudriavzevii*, *S. uvarum* and *S. cerevisiae*. Each box and whisker plot depicts the results of experiments concerning one species and one temperature but involving multiple strains (see Table S4 for primary data). Boxes are delimited by the upper (75^th^) and lower (25^th^) percentiles of the experimental values and the horizontal line inside the box denotes the median of the complete data set. Minimum and maximum values are shown as asterisks and the mean value as a small open square.

Changes in the performances of glycolytic enzymes at different temperatures are only expected to have an effect on organismal fitness if they have an impact on the overall glycolytic flux. Therefore, we measured the glycolytic flux at different temperatures in several strains of *S. cerevisiae* (thermotolerant), *S. kudriavzevii* and *S. uvarum* (both cryotolerant). Between two and six strains were tested per species in order to be able to distinguish species-specific trends from strain-specific effects which are much less likely to be related with the pattern of protein evolution. The glycolytic flux and respiratory activity were measured with a Warburg volumetric apparatus, which allows the quantification of produced CO_2_ and consumed O_2_ during metabolization of glucose by resting cells, at a set temperature [57]. As expected, we found some intra-specific variation in the glycolytic fluxes measured (Figure 4; Table S4). However, it was also possible to unravel species-specific trends. At 10°C, *S. kudriavzevii* exhibited significantly (p<0.0001) higher glycolytic flux than *S. cerevisiae* (Figure 4). Notably, the higher glycolytic flux in *S. kudriavzevii* is accompanied by the lowest respiratory activity of all three species (p<0.0001), which means that the increased flux is strongly directed to ethanol production. On the contrary, the other cryotolerant species, *S. uvarum*, seems to have a higher respiratory capacity than *S. cerevisiae* at 10°C (p = 0.02), and clearly higher than *S. kudriavzevii* (p<0.0001), but an intermediate glycolytic flux.

The glycolytic flux at higher temperatures was subsequently measured at 30°C and at 35°C (Figure 4). In *S. cerevisiae*, the flux at 35°C is higher than at 30°C (p = 0.0006), implying that all the enzymes of the glycolytic pathway are sufficiently heat resistant to cope with this temperature (Figure 4). On the contrary, at 35°C the glycolytic flux does not increase in *S. uvarum*. For *S. kudriavzevii*, there may be even a small decrease of the glycolytic flux at 35°C when compared to 30°C (p = 0.09) (Figure 4). Notably, *S. uvarum* and *S. kudriavzevii* exhibit similar glycolytic rates but very different growth rates at these higher temperatures: while *S. kudriavzevii* grows very poorly already at 30°C [25], and fails to grow at 35°C, one of the *S. uvarum* strains tested (CBS 7001) exhibited similar growth rates at 30° and 25°C (results not shown). Taken together, these results suggest that the failure of *S. kudriavzevii* to grow well at temperatures above 30°C does not seem to be related with a limitation at the level of the glycolysis but that another process essential for growth is compromised at higher temperatures. Hence, our results suggest that evolution of the glycolytic pathway lead in *S. kudriavzevii* to a better performance at lower temperatures, possibly concomitant with a loss of performance at higher temperatures, which nevertheless does not seem to be the growth limiting factor for this species at 30°C.

Contrary to what was observed at 10°C, there was no clear difference between respiration rates in *S. uvarum* and *S. cerevisiae* at 30° and 35°C (Figure 4).

### Temperature adaptation of the first steps of glycolysis

We next considered the activity of individual steps of glycolysis. To ascertain if species-specific trends could be uncovered for independent glycolytic reactions, enzymatic activities were measured in cell-free extracts of a similar set of *S. cerevisiae*, *S. kudriavzevii* and *S. uvarum* strains used in the experiments described in the previous section.

The glucose phosphorilation step in glycolysis potentially involves three enzymes (Hxk2p, Hxk1p and Glk1p) which are expressed under different growth conditions in *S. cerevisiae* [58]. In the presence of abundant fermentable sugars, only Hxk2p is expressed, while the genes encoding the other two enzymes are repressed. *HXK2* is, in turn, repressed at low sugar concentrations or in the absence of sugar [59]. To evaluate if the pattern of expression was the same in the three *Saccharomyces* species under study, we determined the expression of *HXK1* and *HXK2* by quantitative Real-Time Reverse Transcription PCR in two strains of each species cultivated in the presence of high glucose concentrations. Surprisingly, the results show that *HXK1* is the most expressed gene in the two *S. uvarum* strains, in sharp contrast to the *S. cerevisiae* strains which, as previously observed [60], expressed *HXK2* at much higher levels under these conditions (Figure S3). *S. kudriavzevii* strains also express *HXK1* at higher levels, although *HXK2* is also significantly expressed (Figure S3).

**Figure 5:**
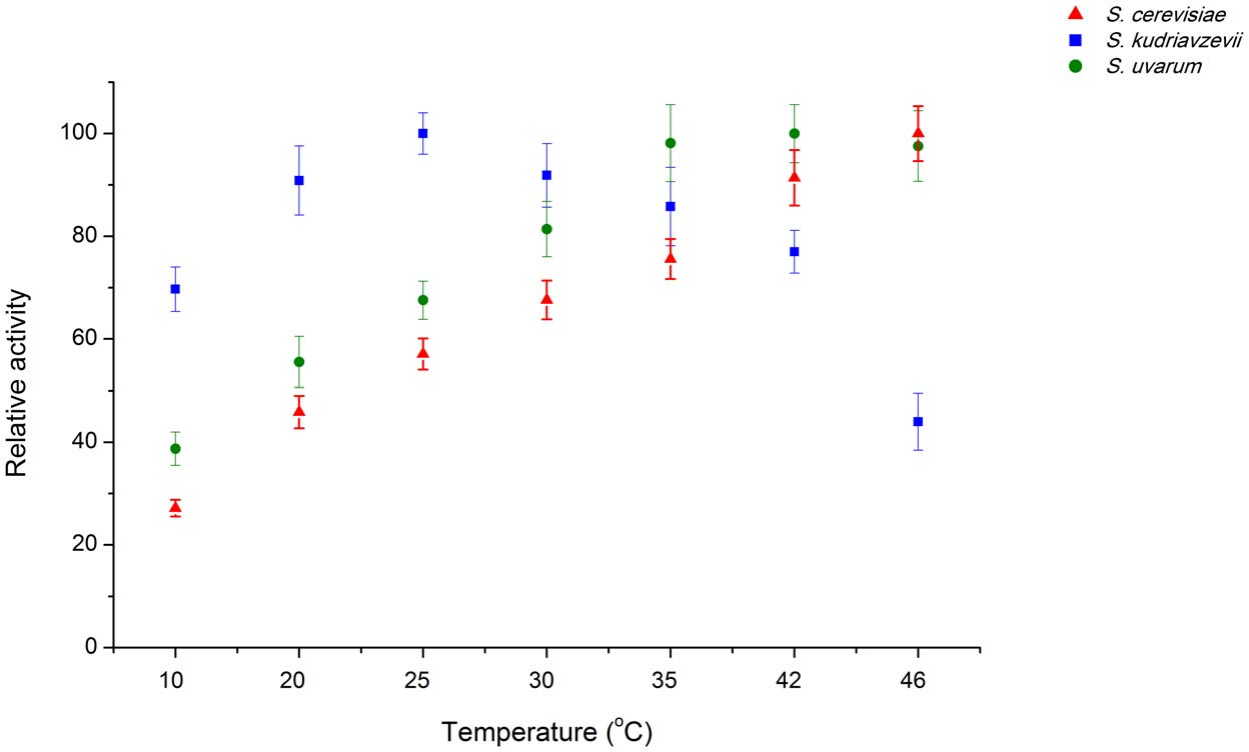
Thermal profile of hexokinase activity in different species. Relative total hexokinase activity measured in *S. cerevisiae*, *S. kudriavzevii* and *S. uvarum* at different temperatures. Activities were measured for each extract at the complete range of temperatures in a single experiment. Each point is the mean value obtained for a species at a given temperature, involving multiple strains and experiments. For each species profile, the highest relative activity measured was set at 100%.

We subsequently determined and compared the temperature profiles of total hexokinase activity in *S. cerevisiae*, *S. kudriavzevii* and *S. uvarum* (Figure 5). Given the different patterns of expression of the *HXK1* and *HXK2* genes in the three species, this activity may correspond in *S. kudriavzevii* to, at least, Hxk1p and Hxk2p, while in *S. cerevisiae* it is attributable only to Hxk2p. We did not determine the pattern of expression of Glk1p in *S. kudriavzevii* and *S. uvarum*, therefore this enzyme may also contribute to the total activity in these two species. In *S. cerevisiae, GLK1* is not expressed in the presence of high glucose concentrations as employed in our experiments [58]. Between two and four strains were tested per species, to be able to distinguish trends associated with each species from strain-specific effects. Hexokinase activity was measured in total protein extracts and absolute activity levels were found to vary, sometimes considerably, among strains of the same species. The most striking variation was observed for *S. cerevisiae* reference strain S288C, which exhibited strongly elevated activities of all glycolytic enzymes tested when compared with all the other strains of the same species (data not shown). Curiously, S288C has been previously shown to exhibit particular and aberrant phenotypes when compared with other *S. cerevisiae* strains which were attributed to differences at the level of transcriptional regulation [61]. In general, we found absolute activities measured in crude extracts to be considerably variable among individual strains of the same species. Therefore, instead of absolute activity levels, we used relative hexokinase activities (for each strain all measurements refer to the highest activity measured) to construct the profiles shown in Figure 5, thereby facilitating comparisons between species.

Like observed for the glycolytic flux, it was possible to highlight species-specific trends in temperature profiles of hexokinase activity (Figure 5). The most prominent species-specific difference occurs at the lower temperature ranges, where *S. kudriavzevii* exhibits a much better performance than both *S. cerevisiae* and *S. uvarum* hexokinases. However, in *S. kudriavzevii* the temperature at which maximum activity is observed (25°C) is much lower than for *S. cerevisiae* and *S. uvarum*. For *S. kudriavzevii*, the higher activities observed at 10°C are in line with the increased glycolytic flux at this temperature but the observed decrease in thermal stability of hexokinase activity in this species does not seem to have a strong effect on the glycolytic flux at 30° and 35°C (Figure 4). This could mean that under these conditions hexokinase activity is not limiting the glycolytic flux.

**Figure 6:**
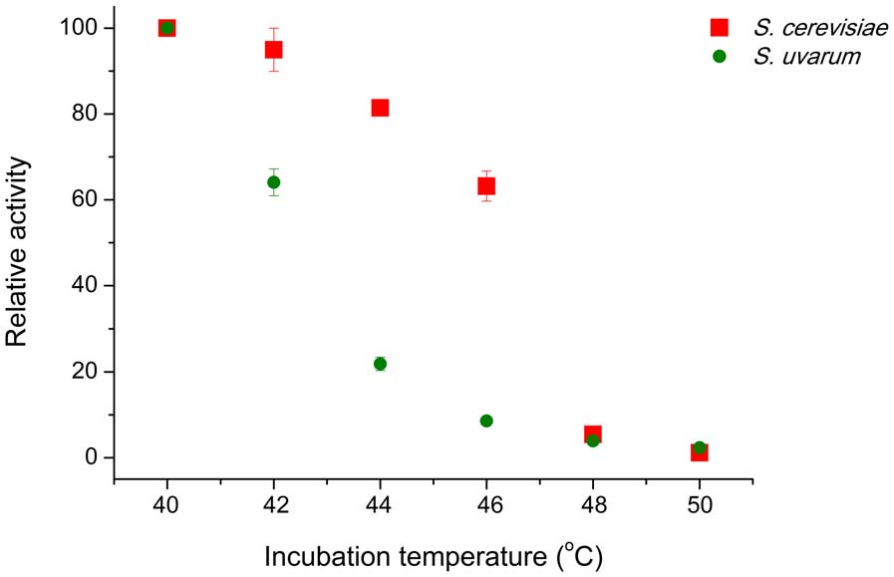
Thermal stability of hexokinase activity in *S. cerevisiae* and *S. uvarum.* Total relative residual hexokinase activity measured in *S.cerevisiae* and *S. uvarum* after preincubation of crude extracts at increasingly higher temperatures for 5 minutes. Points represent mean values. The highest relative activity measured for each strain was set at 100%.

The activity profiles in *S. cerevisiae* and *S. uvarum* differ at the highest temperatures, albeit only slightly, suggesting that the *S. uvarum* hexokinase activity could be more heat sensitive. To test this possibility, a series of assays were performed at 25°C, after pre-incubating the protein extracts at a range of higher temperatures for a short period of time. The results suggest that hexokinase activity in *S. uvarum* is less heat-resistant than in *S. cerevisiae* (Figure 6).

We also determined the temperature profile of the activity of the enzyme that catalyses the second step in glycolysis, phosphoglucoisomerase (PGI; Figure S4). Again, PGI relative activity was higher at the lower temperature range in the *S. kudriavzevii* strain but no loss of activity was observed at higher temperatures. The profiles for the *S. uvarum* and *S. cerevisiae* enzymes were very similar.

### Evidence for divergent selection

Our results support the hypothesis that adaptation to different growth temperatures is likely to have undergone divergent selection along the various *Saccharomyces* lineages. Therefore, it seems plausible that adaptation to different temperature regimes promoted ecological speciation in this genus. In the course of our analysis we found several arguments that favor this possibility. Firstly, genes previously identified as being involved in the adaptation to suboptimal temperatures were found to be among the genes that diverged the most between cryotolerant and thermotolerant species, which favors the possibility that divergent selection determined their evolution pattern. Striking examples of this are the *TIR1* and *TIR4* genes encoding cell wall mannoproteins, which are induced by cold shock [52] and other involve the *ECD1* and *ECD2* genes encoding proteins important for translation during persistent heat stress [62]. Translation elongation factors and protein components of the ribosomal stalk whose role is to recruit the former to the ribosome, are also significantly overrepresented in the group of accelerated genes. Notably, the thermal stability of interactions at the level of the ribosomal stalk have been shown to correlate with growth temperature preferences in bacteria [63], suggesting a requirement for adaptation of this structure to be able to function well at different temperatures. Secondly, the glycolytic pathway, which lighted up under the premises of our analyses as likely to have undergone some form of divergent evolution, was found to exhibit species-specific trends that matched the growth temperature preferences of the species studied. While examining the glucose phosphorilation step in more detail, we observed that the paralogous genes *HXK2* and *HXK1* were very differently regulated in the three species studied, suggesting that the preferred expression of *HXK2* at high glucose concentrations appeared in the lineage leading to *S. cerevisiae.* In this respect, it would be interesting to investigate whether Hxk2p might be the most heat stable of the two enzymes. If this was the case, inversion of the expression pattern might have constituted another contribution to improve the performance of this step of glycolysis at higher temperatures. However, it is also important to note that Hxk2p has a fundamental regulatory role in the main glucose repression pathway in addition to its role in glycolysis [54]. In *S. cerevisiae* this role is specific for Hxk2p, since Hxk1p is able to take over the regulatory role of Hxk2p only when overexpressed [64]. This means that there is some incipient subfunctionalization between the paralogues which is likely to be relevant for the evolution of the two genes. Presumably, Hxk1p is performing the regulatory function in *S. uvarum* where the main glucose repression pathway is thought to operate similarly to *S. cerevisiae*.

### *S. kudriavzevii* and *S. uvarum*: different strategies for cold tolerance

Our *in silico* analysis was focused on the cryotolerant / thermotorelant pair *S. uvarum/S. cerevisiae* because the quality of the available sequences is much better for *S. uvarum* than for *S. kudriavzevii*, the other cryotolerant species [41], [42]. However, a more focused analysis concerning only glycolytic genes showed that in the comparison involving *S. kudriavzevii,* several glycolytic genes exhibited significantly higher dN/dS ratios (Table S3). Regardless of this, it should be noted, that the cryotolerant phenotypes of *S. uvarum* and *S. kudriavzevii* seem to be either the result of convergent evolution or of persistence in both species of an ancestral preference for growth at lower temperatures. In fact, it is consensual that the two species are not phylogenetically close [47] and we showed that carbon and energy metabolism respond very differently to temperature variations in the two species. While *S. uvarum*, a species that is closer to the root of the *Saccharomyces* tree (Figure 1), exhibits improved respiration rates at low temperatures, *S. kudriavzevii* seems to have specialized in ethanol production in similar conditions. Also, the glycolytic genes examined are not more similar between *S. uvarum* and *S. kudriavzevii* than in the comparison of each species with *S. cerevisiae.*

### A hypothesis for the evolution of growth temperature preferences in *Saccharomyces*

In *S. cerevisiae*, there seems to be no clear rate limiting step in the conversion of intracellular glucose to pyruvate [65]. Therefore, the control over the glycolytic flux is in most conditions considered to be shared by all the enzymes. We found that approximately half of the glycolytic genes displayed accelerated dN/dS ratios when species with dissimilar growth temperature preferences were compared. We envisage that the glycolytic genes for which such accelerated evolution is not observed may encode enzymes whose eventual loss of performance at suboptimal temperatures did not compromise the total glycolytic flux (and fitness) in the course of evolution, because they did not become rate limiting upon that loss of activity. Taken together, and superimposed with the phylogenetic relationships depicted in Figure 1, our results support the view that the ancestral *Saccharomyces* phenotype may have resembled that of *S. uvarum* in what concerns the performance of glycolysis. This view is also supported by a recent study examining evolution of growth temperature preferences in *Saccharomyces* employing different approaches [66]. The lineage leading to *S. cerevisiae* seems to have evolved towards a better performance of the glycolytic pathway at higher temperatures denoted by higher fluxes than the other species above 30°C and a slightly improved thermal resistance of hexokinase activity. Conversely, the lineage leading to *S. kudriavzevii* evolved a glycolytic pathway that performs clearly better at low temperatures, which seems to have occurred at the cost of heat resistance, at least for hexokinase activity. In addition, the emphasis in this species seems clearly to be on ethanol production, which may be of ecological significance.

The *in silico* approach in the present work was guided by a specific working hypothesis concerning the role of growth temperature preference in the evolution of *Saccharomyces,* instead of being guided by phylogeny, like other approaches previously undertaken by others [44]. In short, an approach based on phylogeny might hopefully help to find answers to the broad question of which traits were under adaptive selection during the evolution of *Saccharomyces* species. The simpler, pairwise approach we chose aims to address the specific question of whether we can find evidence to support the hypothesis that growth temperature was an important trait during *Saccharomyces* evolution. It is likely that several relevant gene categories may have been missed in our analyses mainly because elevated dN/dS ratios may be confined to a functionally highly significant region of the gene that is nevertheless too short to impart a higher than average global dN/dS ratio to the entire gene. Growth temperature preference is known to be a highly complex phenotype [67]. Therefore, we anticipate that complementary experimental and *in silico* approaches will be able to uncover more genes related to this trait, thereby facilitating investigation of its relevance in a likely ecological speciation scenario in *Saccharomyces*.

## Materials and Methods

### Bioinformatics

The ORFeomes for *S. cerevisiae, S. uvarum, S. kudriavzevii and S. mikatae* were obtained as multi FASTA files from the SGD (http://www.yeastgenome.org/) FTP server. Annotations of different quality resulted in the retrieval of different numbers of ORFs for the various species: *S. cerevisiae*, 6718 ORF; *S. paradoxus*, 6372 ORF; *S. mikatae*, 6228 ORF; *S. kudriavzevii*, 5785 ORF; *S. uvarum*, 6185 ORF. A small number of *S. kudriavzevii* ORFs was discarded during the analysis due to the presence of intervening STOP codons. Pairs of ORFeomes were subsequently chosen and subjected to bi-directional BLAST [68] in order to assess every homologous ORF according to our parameters. As a filter, rather than a specific e-value threshold, we relied on the extension of the global alignment. Bi-directional BLAST was carried out using the BiDiBlast Java pipeline [49]. The pipeline chains the reciprocal Blast with the refinement of every hit through global alignment of the putative homologous ORFs. Only ORFs aligning all their extension within 10% tolerance were retained as valid homologues. Reciprocal hits were tentatively classified as orthologs, and unidirectional hits were assigned to paralogs. The global alignment served also to infer the degree of similitude between homologous ORFs. The BiDiBlast pipeline calculates dN/dS ratios [69] over a codon-wise alignment of the matched ORFs. The complete set of results was uploaded into a relational database, and queries were built either to filter the data or to explore the relationships of interest.

The functional assignment of genes exhibiting unusually high dN/dS ratios in the *S. uvarum/S. cerevisiae* comparison, when compared with the *S. paradoxus/S. cerevisiae* comparison were manually purged from residual artifacts resulting from incompleteness or poor quality of the sequences and the functional/structural categories significantly enriched were essentially manually identified. Functional/structural groups “Ribosomal stalk proteins”, “Translation elongation factors” and “Cell wall mannoproteins” were manually assembled based on gene descriptions at SGD ; the category “glycolysis” was defined as in Conant & Wolfe [39]. The genes included in each category are listed in Table S2. The SGD Gene Ontology Slim Mapper was subsequently used to help highlight functional categories that might be enriched in the outlier group but had escaped the manual examination, but no additional functional categories were identified. Binomial tests(http://www.graphpad.com/quickcalcs/binomial1.cfm) were performed to assess statistical significance of the enrichment of functional categories considering that the total number of ORFs retained in the analysis was 3583 and the number of outliers, defined as points significantly different from the main trend with a higher dN/dS ratio in the *S. uvarum/S. cerevisiae* comparison was 359. In order to detect outliers significantly different from the main trend the standard error about the linear regression parameters was used. Because the trend was very well defined, and the amount of points was very large, the estimate for the uncertainty about the regression line was low. We considered that this estimate was not useful because the observed dispersion of the points around the line was not even, and it seemed to grow along that same line. In order to account for this effect we added this trend to the uncertainty derived from the linear regression parameters. Moreover, the residual distribution parameters were affected by the t Student value when the significance level is 0.05. Thus, the resulting dashed lines in Figure 3 provide a stringent criterion to define the outlier group.

### Competition assays

Two pairs of sympratic strains: *Saccharomyces kudriavzevii* ZP 591 *vs S. cerevisiae* ZP 567 and *S. uvarum* ZP 555 *vs S. paradoxus* ZP 551 were used to perform competition assays in batch cultures at different temperatures (5, 10, 15, 20, 27 and 30°C). The strains were inoculated separately in YNB with 1% glucose and incubated at the given temperature. At the beginning of the exponential growth phase, the two cultures were mixed in approximately equal proportions and a sample of the mixture was immediately harvested for DNA isolation (initial sample). The mixed cultures were subsequently incubated at the set temperature and harvested at stationary phase (final sample). Genomic DNA was extracted from initial and final samples as previously described by Soni & Murray [70], with a few modifications. Briefly: cells were harvested and resuspended in 500 µl lysis buffer (10 mM Tris-HCl pH 8.0, 100 mM NaCl, 1 mM EDTA (pH 8.0), 2% Trinton X-100, 1% SDS). An equal volume of phenol/chloroform (1∶1 v/v) was subsequently added and the whole mixture was vigorously shaken on a Ika-Vibrax VXR shaker at 1800 rpm for 20 minutes, at room temperature and centrifuged at 14000 rpm for 20 minutes at 4°C. Nucleic acids were precipitated with ethanol. The relative proportions of the competing strains in the initial and final samples was determined by qRT- PCR using the TAQurateTM GREEN Real-Time PCR MasterMix (Epicentre), each 12.5 µl reaction containing 0.1 µM of each primer (Table S1B) and 50 ng genomic DNA. Amplification consisted of an initial denaturation step at 94°C for 3 min, followed by 40 cycles of 30 s at 94°C, 30 s at variable annealing temperature (see Table S1B) and 30 s at 72°C. PCR was performed in a lightcycler Rotor-Gene 6000 apparatus (Qiagen, Germany). Reactions were performed in triplicate. The efficiency of the four PCR reactions was determined using pure genomic DNA of each of the competing strains and was used to calculate the relative amount of genomic DNA of each competing strain in the initial and final samples of each culture.

### Quantification of *HXK1* and *HXK2* expression using qRT-PCR

*HXK1* or *HXK2* primers (Table S1C) allowed specific amplification of regions of the *HXK1* and *HXK2* spanning part of the coding regions in all the *Saccharomyces* species under study. Total RNA was isolated using TRIzol® (Invitrogen, Carlsbad, USA) according to the manufacturer’s instructions. The cultures were grown in 100 ml YPD (1% yeast extract, 2% peptone, 2% glucose) at 25°C , 150 rpm until O.D._640 nm_ 0.8 and were collected and frozen in liquid nitrogen. RNA samples were subsequently treated with DNaseI (Qiagen, Germany) and submitted to a conventional PCR reaction using the *HXK1* and *HXK2* primer pairs, to check for the absence of genomic DNA contamination. qReal-Time RT-PCR reactions were performed with MasterAmpTM GREEN Real-Time RT-PCR Kit (Epicentre), each 12.5 µl reaction containing 0.5 mM MnSO_4_, 0.1 µM of each primer (Table S1C), 40 ng/reaction of purified RNA and 2.5U RetroAmp RT DNA polymerase Amplification consisted of an initial RT- step at 60°C for 30 min, followed by a denaturation step at 94°C for 3 min, then by 40 cycles of 30 s at 94°C, 30 s at 49°C and 30 s at 72°C. For each RNA sample at least two independent qReal-Time RT-PCR experiments were carried out, usually in triplicate. The efficiency of each reaction was calculated using genomic DNA and were used to determine the relative amounts of *HXK1* and *HXK2* mRNAs.

### Crude extracts for enzymatic assays

The cultures were grown in YPD (1% yeast extract, 2% peptone, 2% glucose) at 25°C, 150 rpm until mid exponential phase and were harvested by centrifugation (4°C, 10 min at 9000 rpm), washed twice with phosphate buffer (10 mM potassium phosphate, 2 mM EDTA pH 7.5), concentrated fourfold, and stored at −80°C. Before assaying, the cells were thawed, washed and resuspended in 400 µl of phosphate buffer with 2 mM MgCl_2_ and 1 mM DTT. Lysis buffer (0.1 M potassium phosphate, 2 mM MgCl_2_ and 1 mM DTT) and 200 µl glass beads (∅ 230–320 nm) were subsequently added to the suspension. The cells were disrupted by six alternating cycles of 1 min vortexing followed by 1 min cooling on ice. Cell debris were removed by centrifugation (4°C, 20 min at 13000 rpm), and the supernatant was immediately used for enzymatic assays. Total protein concentration in the crude extracts was determined using the BCA protein assay kit (PIERCE) with bovine serum albumin as a standard.

### Enzymatic assays

Enzymatic assays were always performed using freshly prepared crude extracts of the following strains: *S. cerevisiae*, S288C; CEN.PK113-11C, ZP 567 and ZP 736; *S. kudriavzevii*, IFO 1802, ZP 591, ZP 513 and ZP 828; *S.uvarum*, CBS 7001, ZP 555 and ZP 663.

Hexokinase (HXK; EC 2.7.1.1) was assayed in 5 mM glucose, 1 mM ATP, 0.25 mM NADP^+^, and 0.54 unit of glucose-6-P dehydrogenase and phosphoglucose isomerase (PGI; EC 5.3.1.9) in 1 mM fructose-6-P, 0.05 mM NADP^+^, and 0.27 unit of glucose-6-P dehydrogenase, both in 1 ml reaction volume. The rate of the reactions was determined by measuring the absorbance at 340 nm using an UV-2101PC thermostatized spectrophotometer (Shimadzu, Japan) for 1 minute after the reaction was started. The reactions were always started by the addition of sugars, after pre-incubation of the extract at the assay temperature for 10 sec. All assays were performed using three different amounts of cell extracts (5; 7.5 and 12.5 µg protein).

To determine the relative thermal stability of hexokinase activity in *S. uvarum* and *S. cerevisiae,* a sample of the crude extract was pre-incubated at 40, 42, 44, 46, 48 and 50°C for 5 min, and immediately cooled on ice. The amount of residual enzyme activity was subsequently assayed at 25°C.

### Warburg Assays

Cultures were grown in 100 ml YNB with 2% glucose at 25°C, 150 rpm until mid exponential phase, harvested by centrifugation (4°C, 10 min at 9000 rpm), washed twice with phosphate buffer (100 mM potassium phosphate, pH 4.5), and resuspended in the appropriate volume of buffer to a final concentration of approximately 1 mg dW/ml.

Glucose consumption was evaluated in resting cells using a Warburg volumetric apparatus (B. Braun Melsungen), which allows the quantification of CO_2_ produced and O_2_ consumed [57]. At least two different cultures were performed for each strain and each measurement was performed in duplicate. The complete set of results is summarized in Table S4. Statistical significance of the differences observed between datasets was assessed using an unpaired T-test (http://www.graphpad.com/quickcalcs/ttest1.cfm), reported *p* values are two tailed.

## Supporting Information

Figure S1 **Outcome of competition between sympatric** ***Saccharomyces*** **species.** Relative fitness of *S. paradoxus* ZP 551 competing with *S. uvarum* ZP 555 at different temperatures is shown. The relative size of the populations of both species was determined in the beginning and at the end of each co-culture experiment by qRT-PCR using species specific primers and was used to calculate the relative fitness of each species.
(PDF)

Figure S2 **Comparison of dN/dS ratios of** ***Saccharomyces*** **orthologous gene pairs in different species comparisons.** dN/dS ratios depicted were determined between *S. uvarum* and *S. cerevisiae* (Yaxis) and between *S. paradoxus* and *S. cerevisiae* (Xaxis). Each point corresponds to one orthologous gene pair that could be reliably identified in both species comparisons. The regression line is shown in solid blue. The dashed lines add the contribution of the residuals distribution. Points outside these lines may be considered as significantly different from the general trend. The group of orthologs of interest to the present analysis displays higher dN/dS ratios in the comparison between the *S. uvarum* and S. *cerevisiae* genomes than in the comparison between *S. paradoxus* and S. *cerevisiae*, corresponding therefore to data points located above the segment defined by the dashed lines. Points representing glycolytic genes are highlighted as red circles. Primary data used to construct the plot is listed in Table S2.
(PDF)

Figure S3 Relative expression of the *HXK1* (grey) and *HXK2* (black) genes determined by RT-Real Time PCR in six strains belonging to *Saccharomyces cerevisiae, S. uvarum* and *S. kudriavzevii*, as indicated.
(PDF)

Figure S4 **Thermal profile of phosphoglucose isomerase (PGI) activity in different species.** Relative total phosphoglucose isomerase activity measured in *S. cerevisiae* CEN.PK-113-11C, *S. kudriavzevii* IFO 1802^T^ and *S. uvarum* CBS 7001 at different temperatures. Activities were measured for each extract at the complete range of temperatures in a single experiment. Each point is the mean value of the data obtained for each strain at the given temperature. For each strain profile, the highest relative activity measured was set at 100%.
(PDF)

Table S1 **A.** List of strains used in this study. **B.** Primers and annealing temperatures used for Real-Time PCR analyses in competition assays (Fig. 2 and Fig. S1). **C.** Primers and annealing temperatures used for RT-Real-Time PCR analyses of the relative expression of the *HXK1* and *HXK2* genes (Fig. S3).
(PDF)

Table S2 dN, dS and dN/dS ratios determined for *Saccharomyces* ORFeomes between *S. paradoxus* and *S. cerevisiae* and between *S. uvarum* and *S. cerevisiae*.
(XLS)

Table S3 dN/dS ratios determined for glycolytic genes in different *Saccharomyces* species comparisons.
(PDF)

Table S4 Glycolytic and respiratory fluxes measured in Warburg assays.
(PDF)
