# Supplementary material for: Evidence for Divergent Evolution of Growth Temperature Preference in Sympatric Saccharomyces Species
Source: PLoS One. 2011 Jun 2;6(6):e20739. doi: 10.1371/journal.pone.0020739 (PMC3107239; doi:10.1371/journal.pone.0020739)

Figure S1. Outcome of competition between sympatric *Saccharomyces* species. Relative fitness of *S. paradoxus* ZP 551 competing with *S. uvarum* ZP 555 at different temperatures is shown. The relative size of the populations of both species was determined in the beginning and at the end of each co-culture experiment by qRT-PCR using species specific primers and was used to calculate the relative fitness of each species.

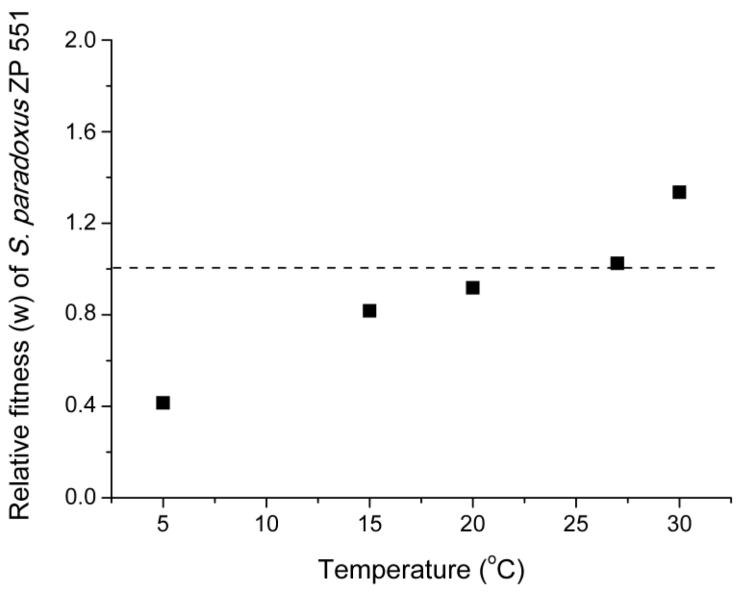

Supplement: Figure S1 — Outcome of competition between sympatric Saccharomyces species. Relative fitness of S. paradoxus ZP 551 competing with S. uvarum ZP 555 at different temperatures is shown. The relative size of the populations of both species was determined in the beginning and at the end of each co-culture experiment by qRT-PCR using species specific primers and was used to calculate the relative fitness of each species. (PDF) [file pone.0020739.s001.pdf]
