# Supplementary material for: Evidence for Divergent Evolution of Growth Temperature Preference in Sympatric Saccharomyces Species
Source: PLoS One. 2011 Jun 2;6(6):e20739. doi: 10.1371/journal.pone.0020739 (PMC3107239; doi:10.1371/journal.pone.0020739)

Figure S2. Comparison of dN/dS ratios of *Saccharomyces* orthologous gene pairs in different species comparisons. dN/dS ratios depicted were determined between *S. uvarum* and *S. cerevisiae* (Yaxis) and between *S. paradoxus* and *S. cerevisiae* (Xaxis). Each point corresponds to one orthologous gene pair that could be reliably identified in both species comparisons. The regression line is shown in solid blue. The dashed lines add the contribution of the residuals distribution. Points outside these lines may be considered as significantly different from the general trend. The group of orthologs of interest to the present analysis displays higher dN/dS ratios in the comparison between the *S. uvarum* and S. *cerevisiae* genomes than in the comparison between *S. paradoxus* and S. *cerevisiae*, corresponding therefore to data points located above the segment defined by the dashed lines. Points representing glycolytic genes are highlighted as red circles. Primary data used to construct the plot is listed in Table S2.

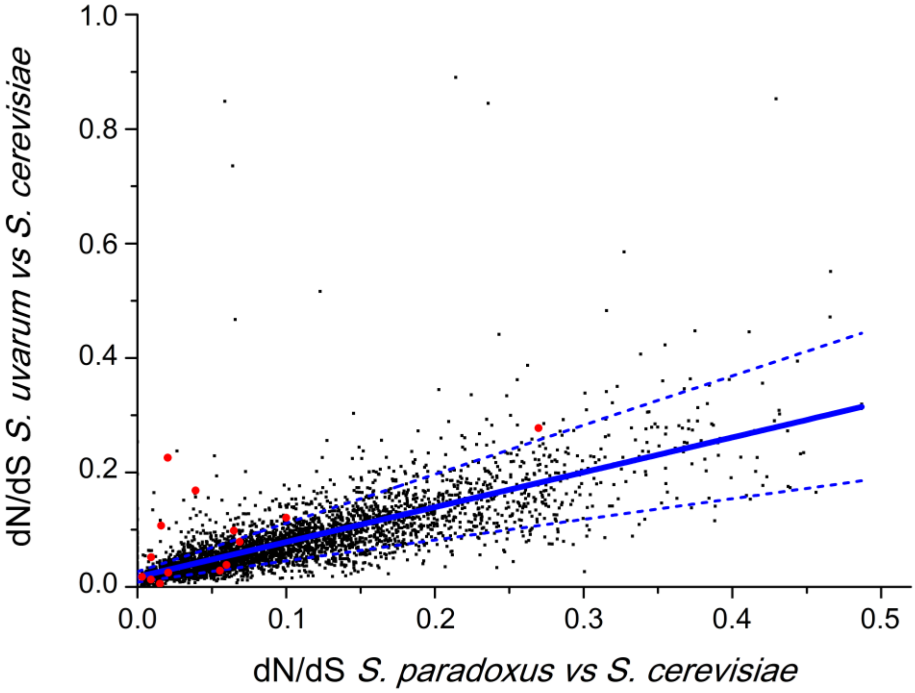

Supplement: Figure S2 — Comparison of dN/dS ratios of Saccharomyces orthologous gene pairs in different species comparisons. dN/dS ratios depicted were determined between S. uvarum and S. cerevisiae (Yaxis) and between S. paradoxus and S. cerevisiae (Xaxis). Each point corresponds to one orthologous gene pair that could be reliably identified in both species comparisons. The regression line is shown in solid blue. The dashed lines add the contribution of the residuals distribution. Points outside these lines may be considered as significantly different from the general trend. The group of orthologs of interest to the present analysis displays higher dN/dS ratios in the comparison between the S. uvarum and S. cerevisiae genomes than in the comparison between S. paradoxus and S. cerevisiae, corresponding therefore to data points located above the segment defined by the dashed lines. Points representing glycolytic genes are highlighted as red circles. Primary data used to construct the plot is listed in Table S2. (PDF) [file pone.0020739.s002.pdf]
