# Supplementary material for: Evidence for Divergent Evolution of Growth Temperature Preference in Sympatric Saccharomyces Species
Source: PLoS One. 2011 Jun 2;6(6):e20739. doi: 10.1371/journal.pone.0020739 (PMC3107239; doi:10.1371/journal.pone.0020739)

Figure S3. Relative expression of the *HXK1* (grey) and *HXK2* (black) genes determined by RT-Real Time PCR in six strains belonging to *Saccharomyces cerevisiae, S. uvarum* and *S. kudriavzevii*, as indicated.

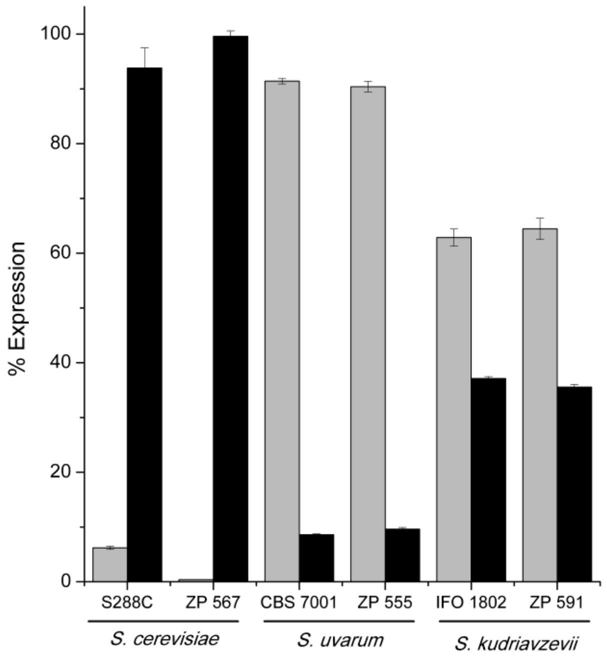

Supplement: Figure S3 — Relative expression of the HXK1 (grey) and HXK2 (black) genes determined by RT-Real Time PCR in six strains belonging to Saccharomyces cerevisiae, S. uvarum and S. kudriavzevii, as indicated. (PDF) [file pone.0020739.s003.pdf]
