# Supplementary material for: Evidence for Divergent Evolution of Growth Temperature Preference in Sympatric Saccharomyces Species
Source: PLoS One. 2011 Jun 2;6(6):e20739. doi: 10.1371/journal.pone.0020739 (PMC3107239; doi:10.1371/journal.pone.0020739)

Figure S4. Thermal profile of phosphoglucose isomerase (PGI) activity in different species. Relative total phosphoglucose isomerase activity measured in *S. cerevisiae* CEN.PK-113-11C, *S. kudriavzevii* IFO 1802^T and *S. uvarum* CBS 7001 at different temperatures. Activities were measured for each extract at the complete range of temperatures in a single experiment. Each point is the mean value of the data obtained for each strain at the given temperature. For each strain profile, the highest relative activity measured was set at 100%.

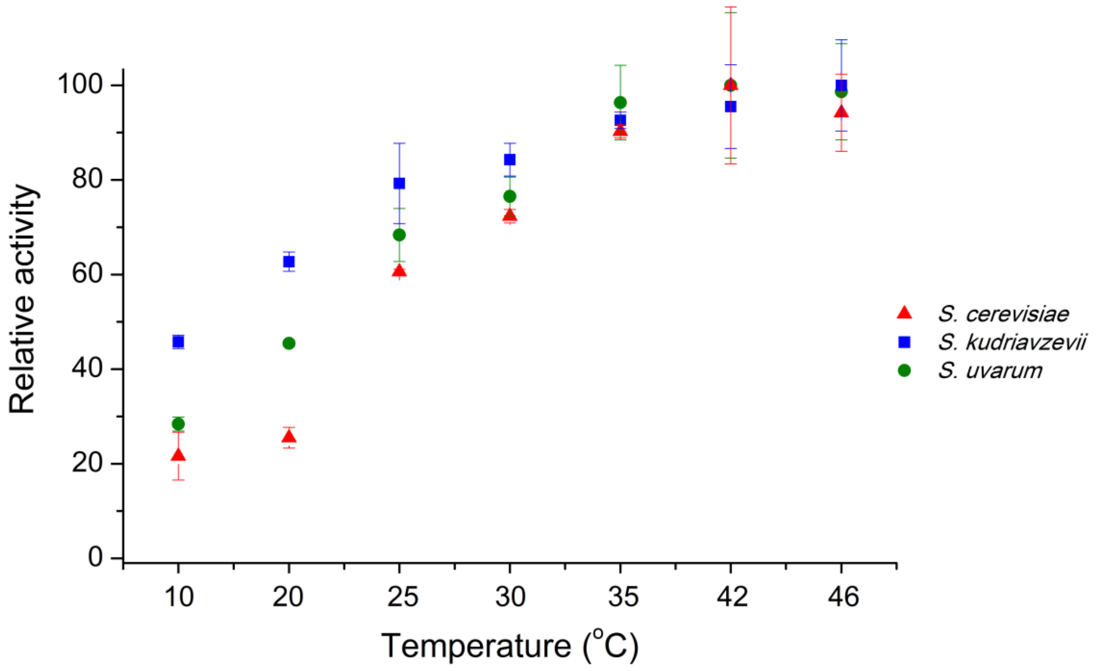

Supplement: Figure S4 — Thermal profile of phosphoglucose isomerase (PGI) activity in different species. Relative total phosphoglucose isomerase activity measured in S. cerevisiae CEN.PK-113-11C, S. kudriavzevii IFO 1802T and S. uvarum CBS 7001 at different temperatures. Activities were measured for each extract at the complete range of temperatures in a single experiment. Each point is the mean value of the data obtained for each strain at the given temperature. For each strain profile, the highest relative activity measured was set at 100%. (PDF) [file pone.0020739.s004.pdf]
