# Supplementary material for: Evidence for Divergent Evolution of Growth Temperature Preference in Sympatric Saccharomyces Species
Source: PLoS One. 2011 Jun 2;6(6):e20739. doi: 10.1371/journal.pone.0020739 (PMC3107239; doi:10.1371/journal.pone.0020739)
Supplement: Table S1 — A. List of strains used in this study. B. Primers and annealing temperatures used for Real-Time PCR analyses in competition assays (Fig. 2 and Fig. S1). C. Primers and annealing temperatures used for RT-Real-Time PCR analyses of the relative expression of the HXK1 and HXK2 genes (Fig. S3). (PDF) [file pone.0020739.s005.pdf]

Table S1A. List of strains used in this study.

Species	Strain	Isolation source	Remarks
Saccharomyces cerevisiae	S288C (CBS 8803)		Reference genome
	PYCC 5485		CEN.PK-113-11C
	ZP 567	Bark of *Q. pyrenaica*, Castelo de Vide, Portugal	Sampaio & Gonçalves, AEM 2008
	ZP 736	Rotten Figs, Abrantes, Portugal	
	ZP 805	Bark of *Quercus glauca*, Takamatsu-shi, Kagawa pref., Japan	
	ZP 851	Soil underneath *Q. illex*, Alconorales Natural Park, Andaluzia, Spain	
	ZP 1008	Soil underneath *Q. faginea*, Eja, Melres, Douro, Portugal	
S. kudriavzevii	IFO 1802 (CBS 8840)	Decayed leaf of plant, Japan	Reference genome
	ZP 591	Bark of *Q. pyrenaica*, Castelo de Vide, Portugal	Sampaio & Gonçalves, AEM 2008<br>Sympatric pair of ZP 567
	ZP 513	Bark of *Q. pyrenaica*, Lisboa, Portugal	
	ZP 828	Bark of *Quercus glauca*, Takamatsu-shi, Kagawa pref., Japan	Sympatric pair of ZP 805
	ZP 856	Soil underneath *Q. illex*, Alconorales Natural Park, Andaluzia, Spain	Sympatric pair of ZP 851
	ZP 1009	Soil underneath *Q. faginea*, Eja, Melres, Douro, Portugal	Sympatric pair of ZP 1008
S. uvarum	CBS 7001	*Mesophylax adopersus* (insect), Avila, Spain	Reference genome (*S. bayanus* at SGD)
	ZP 555	Bark of *Q. garryana*, Hornby Island of British Columbia, Canada	Sampaio & Gonçalves, AEM 2008
	ZP 663	Bark of *Q. robur*, Bochum, Germany	
S. paradoxus	ZP 551	Bark of *Q. garryana*, Hornby Island of British Columbia, Canada	Sampaio & Gonçalves, AEM 2008<br>Sympatric pair of ZP 555

Table S1B. Primers and annealing temperatures used for Real-Time PCR analyses in competition assays (Fig. 2 and Fig. S1).

Species	Primer name	Sequence	Annealing Temperature
S. kudriavzevii	SRC_Met6_Fw	AACAATTRTTGCCTCTATACRCTGAAA	49ºC
	SRC_Met6_RevSk	ATAAAGTTTGCTTAGCACCGATGG	
S. cerevisiae	SRC_Met6_Fw	AACAATTRTTGCCTCTATACRCTGAAA	49ºC
	SRC_Met6_RevSc	ACAAGGTTTGTTTGTTACCAATGG	
S. uvarum	FSY1300For	GTGTCGGCTATCAAATWAACTT	53ºC
	FSY1650Rev	AAGGCAAACAYGTAAAGCAAAG	
S. paradoxus	ActSpSu-F	TACTCTTTCTCCACCACTGC	53ºC
	ActSpSu-R	ACGATGTTACCGTATAATTCCT	

Table S1C. Primers and annealing temperatures used for RT-Real-Time PCR analyses of the relative expression of the *HXK1* and *HXK2* genes (Fig. S3).

Gene	Primer name	Sequence *	Annealing temperature
HXK1	Hxk1for	AGATGGGTGTGATTTTCG	49ºC
	Hxk1rev	AATTCACCCAAGTAGTAACC	
HXK2	Hxk2for	AAACCACAAGCCAGAAA	49ºC
	Hxk2rev	TTTGAGTAGTTCTCATAGC	
