# Supplementary material for: Evidence for Divergent Evolution of Growth Temperature Preference in Sympatric Saccharomyces Species
Source: PLoS One. 2011 Jun 2;6(6):e20739. doi: 10.1371/journal.pone.0020739 (PMC3107239; doi:10.1371/journal.pone.0020739)
Supplement: Table S3 — dN/dS ratios determined for glycolytic genes in different Saccharomyces species comparisons. (PDF) [file pone.0020739.s007.pdf]

Table S3. dN/dS ratios determined for glycolytic genes in different *Saccharomyces* species comparisons.

**Gene**	**dN/dS Spar/Scer**	**dN/dS Suva/Scer**	**dN/dS Skud/Scer**	**dN/dS Smik/Scer**
**HXK1**	0.060	0.038	0.037	0.031
**HXK2**	0.009	**0.052**	0.040	0.021
**GLK1**	0.015	0.006	0.009	0.009
**PGI1**	0.003	0.018	0.028	0.006
**PFK1**	0.009	0.013	0.006	0.019
**PFK2**	0.021	0.025	0.020	0.006
**FBA1**	0.270	**0.278**	0.222	0.205
**TPI1**	0.045	–	**0.115**	–
**TDH1**	0.069	0.079	0.065	0.046
**TDH2**	0.115	–	0.099	0.053
**TDH3**	0.037	–	–	0.069
**PGK1**	0.100	**0.121**	0.066	0.068
**GPM1**	0.020	**0.226**	**0.100**	0.030
**ENO1**	0.016	**0.107**	**0.067**	0.021
**ENO2**	0.039	**0.168**	**0.148**	**0.125**
**CDC19**	0.065	**0.098**	–	0.076
**PYK2**	0.055	0.028	–	0.048

Spar: *S. paradoxus*; **Scer:***S. cerevisiae*; **Skud**: *S kudriavzevii*; **Smik:** *S. mikatae*
–: no ortholog identified
Values shown in red are outliers identified in each species comparison as shown in Figure 3 for *S. cerevisiae/S. uvarum.*
