# Supplementary material for: Evidence for Divergent Evolution of Growth Temperature Preference in Sympatric Saccharomyces Species
Source: PLoS One. 2011 Jun 2;6(6):e20739. doi: 10.1371/journal.pone.0020739 (PMC3107239; doi:10.1371/journal.pone.0020739)
Supplement: Table S4 — Glycolytic and respiratory fluxes measured in Warburg assays. (PDF) [file pone.0020739.s008.pdf]

Table S4. Glycolytic and respiratory fluxes measured in Warburg assays.

Species	Strain	10ºC		30ºC		35ºC	
		Respiration (µl.mg^{-1}.min^{-1})	Total Flux (µl.mg^{-1}.min^{-1})	Respiration (µl.mg^{-1}.min^{-1})	Total Flux (µl.mg^{-1}.min^{-1})	Respiration (µl.mg^{-1}.min^{-1})	Total Flux (µl.mg^{-1}.min^{-1})
S. kudriavzevii	IFO 1802	0.12	1.96	0.63	8.43	0.57	6.04
		0.12	2.08	0.50	6.92	0.38	5.77
	ZP 591	0.15	1.97	0.44	10.31	0.63	8.05
		0.11	2.00	0.64	7.69		
	ZP 513	0.13	1.67				
		0.14	1.70				
	ZP 828	0.15	1.96				
		0.15	2.04				
	ZP 856	0.10	1.45				
		0.11	1.60				
	ZP 1009	0.09	1.52				
S. uvarum	CBS 7001	0.22	1.75	1.35	7.68	1.36	7.47
		0.35	1.69	1.00	6.04	1.26	8.03
		0.29	1.64				
	ZP 555	0.29	1.61	1.21	7.74	0.52	6.62
		0.21	1.42	1.15	6.15	0.92	8.15
	ZP 663			0.90	7.92	0.87	6.63
				0.88	7.48		
S. cerevisiae	CEN.PK-113-11C	0.18	1.22	1.35	8.27	1.30	8.89
		0.17	1.27				
	ZP 567	0.17	1.63	0.84	7.92	1.11	10.90
		0.20	1.59	0.83	7.55	0.72	10.67
		0.19	1.50				
	ZP 736	0.25	1.33	1.81	7.92	1.99	9.62
		0.27	1.30	1.39	6.99	1.94	10.00
	ZP 805	0.17	1.21				
		0.19	1.22				
	ZP 851	0.26	1.32				
		0.25	1.19				
	ZP 1008	0.22	1.29				

Sympatric pairs of strains are shown in the same colors
